# Mediastinal anatomical landmarks, their variants and tips for video-assisted thoracoscopic navigation during oesophageal extirpation

**DOI:** 10.1007/s00276-021-02820-8

**Published:** 2021-08-23

**Authors:** Sergey Dydykin, Friedrich Paulsen, Tatyana Khorobykh, Natalya Mishchenko, Marina Kapitonova, Sergey Gupalo, Tatyana Bogoyavlenskaya, Vadim Agadzhanov, Pashad Salikhov

**Affiliations:** 1 I. M. Sechenov First Moscow State Medical University, Moscow, Russia; 2 Institute of Functional and Clinical Anatomy, Friedrich Alexander University Erlangen-Nürnberg, Erlangen, Germany; 3 Department of Basic Medical Sciences, Faculty of Medicine and Health Sciences, Universiti Malaysia Sarawak, Kota Samarahan, Malaysia; 4 Faculty of Medicine, Bioscience and Nursing, MAHSA University, Bandar Saujana Putra, Malaysia

**Keywords:** Minimally invasive esophagectomy, Anatomic landmarks, Video-assisted thoracic surgery, Thoracoscopy

## Abstract

**Purpose:**

There is no systematic description of primary anatomical landmarks that allow a surgeon to reliably and safely navigate the superior and posterior mediastinum’s fat tissue spaces near large vessels and nerves during video-assisted endothoracoscopic interventions in the prone position of a patient. Our aim was to develop an algorithm of sequential visual navigation during thoracoscopic extirpation of the esophagus and determine the most permanent topographic and anatomical landmarks allowing safe thoracoscopic dissection of the esophagus in the prone position.

**Methods:**

The anatomical study of the mediastinal structural features was carried out on 30 human cadavers before and after opening the right pleural cavity.

**Results:**

For thoracoscopic extirpation of the esophagus in the prone position, anatomical landmarks are defined, their variants are assessed, and an algorithm for their selection is developed, allowing their direct visualization before and after opening the mediastinal pleura.

**Conclusion:**

The proposed algorithm for topographic and anatomical navigation based on the key anatomical landmarks in the posterior mediastinum provides safe performance of the video-assisted thoracoscopic extirpation of the esophagus in the prone position.

## Introduction

Esophageal cancer is among the tumors with the most aggressive and poor survival prognosis, ranking 8th in oncologic morbidity and 6th in cancer mortality worldwide. Despite the advances in chemotherapeutic methods, the basis of the combined treatment of oesophageal cancer is surgery. Currently, increasing attention is being paid to minimally invasive techniques, including a video-assisted thoracoscopic approach [23, 26, 28]. The thoracoscopic approach allows better visualization of the esophagus. Thus, more reliable identification of anatomical structures, which increases the surgical procedure’s safety, facilitates adequate lymphadenectomy, reduces the procedure’s invasiveness and intraoperative blood loss, and decreases the number of postoperative complications. However, typical thoracoscopic surgery complications occur more frequently during the phase of ascending learning curve [14, 29].

It should be stated that video-endoscope-assisted teaching of surgery is currently becoming virtual by nature, as it is out of touch with a surgeon’s training in clinical (topographic) anatomy. The endoscopic surgeon is separated from the patient by a virtual screen when only focused images are visible in the objective, while the integral topographic and anatomical picture of the operated area is missing [9].

Topographic deviation of the posterior mediastinum in the patient’s prone position with an iatrogenically induced collapse of the right lung increases the possibility of accidental damage to critical anatomical structures that run close to the esophagus. Therefore, to safely perform thoracoscopic procedures on the esophagus, it is necessary to highlight the prominent anatomic landmarks that allow the surgeon to reliably and safely navigate through the mediastinal adipose tissue spaces near the great vessels and nerves [2, 3, 5]. However, a systematic description of the essential anatomic landmarks for thoracoscopic navigation during esophageal extirpation is still lacking. Therefore, the determination of these anatomic landmarks served as the basis for this study.

In this regard, the purpose of this study was to develop an algorithm of sequential visual navigation during thoracoscopic extirpation of the esophagus and determine the most durable topographic and anatomic landmarks for safe thoracoscopic mobilization of the esophagus with the patient in the prone position.

## Materials and methods

### Cadavers

Our anatomical study of 30 human cadavers (12 males and 18 females, aged between 32 and 88 with an average age of 74.4 years) was performed at the Department of Pathology, N.V. Sklifosovsky Research Institute of Emergency Medicine, and the Department of Operative Surgery and Topographic Anatomy at Sechenov University.

### Examination procedures

The examination of organs in the posterior mediastinum was performed in the supine position due to technical difficulties in simulating a video-assisted thoracoscopic examination of a cadaver in the prone position.

Classical guidelines on normal anatomy, topographic anatomy, and operative surgery based on modern anatomical terminology were used to identify the main anatomical structures in the posterior mediastinum [12, 19, 22]. The examination was performed in two phases: (1) before and (2) after the opening of the mediastinal pleura.

### Phase one

The collar incision and vertical sternotomy were performed, followed by the anatomical pathology section along the right midclavicular lines, and the sterno-cartilaginous complex was everted.

Before opening the mediastinal pleura, the anatomic and topographic features of the thoracic cavity's organs and structures were examined, including the azygos vein and its arch, the vagus nerve and its branches, the pulmonary ligament, and the azygoaortic sulcus. After performing morphometric measurements (determining the vertebral height of the azygos venous arch and the right recurrent laryngeal nerve loop), the most appropriate anatomical landmarks were identified to determine the topography of adjacent anatomical structures in adipose tissue that were not available for direct visualization [6]. The least variable among the visible external anatomical landmarks were selected. The determined anatomical landmarks were then used in the operative part of the study when performing thoracoscopic esophagectomy and lymphadenectomy.

### Phase two

The second part of the study was performed after opening the mediastinal pleura. Anatomical landmarks determined in the first phase of the study were used as reference points for preparation. The sharp dissection method was used to open the mediastinal pleura. Stratified dissection of the anatomic structures allowed consistent determination of the adjacent anatomic landmarks of the superior and posterior mediastinum: esophagus, thoracic duct, bifurcation of the trachea, main bronchi, aortic arch, superior vena cava, trachea, right recurrent laryngeal nerve, brachiocephalic trunk, and right subclavian artery.

The sequence of manipulations in the mediastinum during intraoperational video-assisted thoracoscopy was as follows.

Mobilization of the thoracic esophagus in the prone position began with transection of the mediastinal pleura from the superior or inferior thoracic aperture, depending on the tumor's location. Its mobilization began from the azygos vein arch if the tumor was localized in the inferior thoracic esophagus. If the tumor was localized in the middle thoracic region, its mobilization began from the pulmonary ligament's inferior edge in the least altered tissue area.

**Fig. 1:**
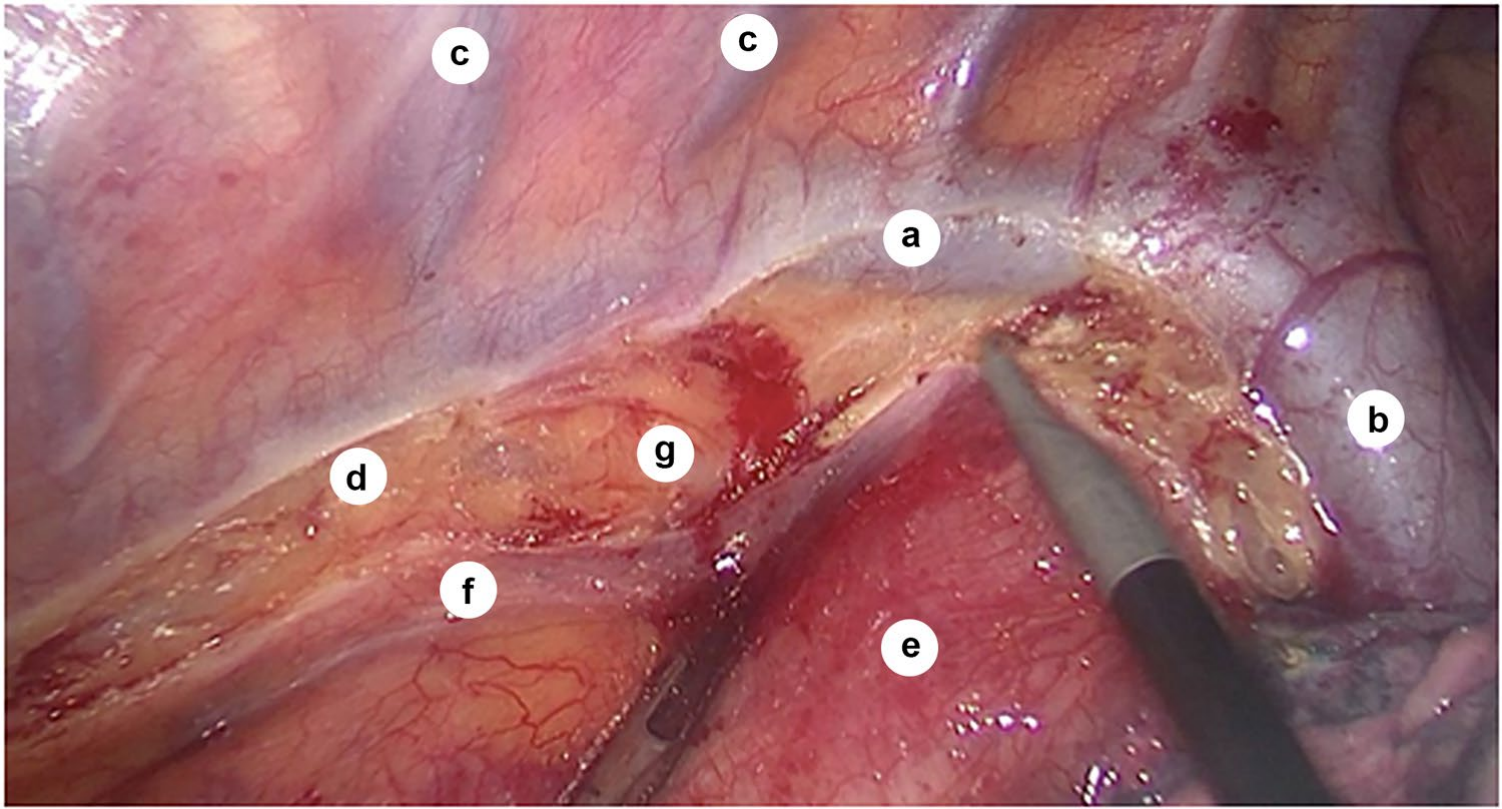
Intraoperative view of the azygos vein towards linea bispinalis; *a*—azygos vein; *b*—arch of azygos vein; *c*—intercostal veins; *d*—sulcus azygoaortalis; *e*—esophagus (under the layer of mediastinal pleura); *f*—mediastinal pleura (after transection); *g*—adventitia of the thoracic aorta

The mediastinal pleura was opened, the azygos vein was mobilized and cut, then clipping was performed to create the confluence of the azygos and hemiazygos veins (Fig. 1).

**Fig. 2:**
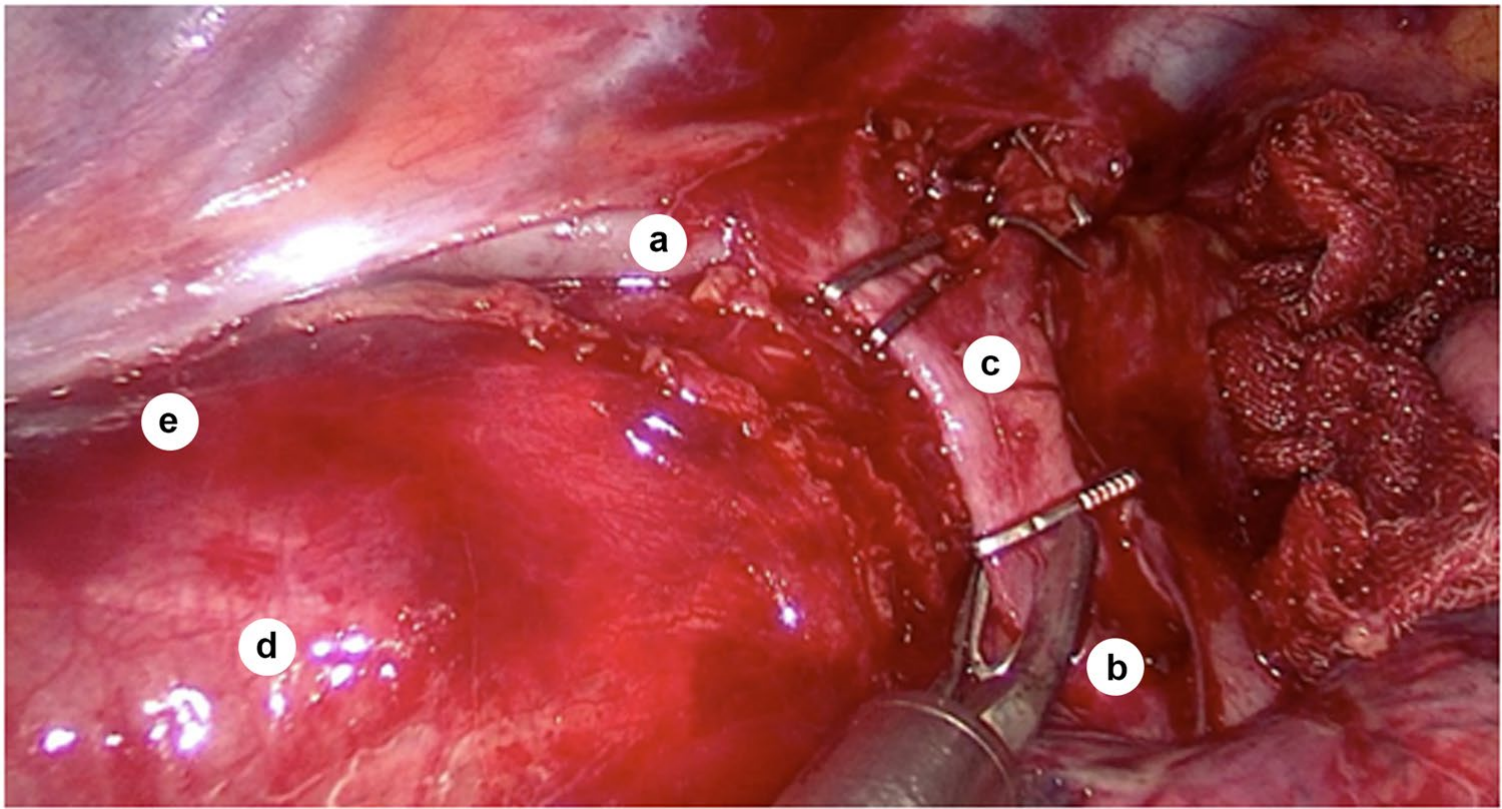
Relationship between azygos and hemiazygos veins during the creation of their confluence (intraoperative photo, sagittal plane); *a*—azygos vein; *b*—hemiazygos vein; *c*—arch of azygos vein; *d*—esophagus (under the mediastinal pleura layer); *e*—sulcus azygoaortalis

Then, access to the bifurcation of the trachea was opened (Fig. 2), and traction of the trunk of the azygos vein allowed lymphadenectomy of the cardiac lymph nodes (group N107), where the left main bronchus forms the left tracheobronchial angle. As the right bronchial artery located under the trunk of the azygos vein could be the only bronchial artery in the body, it should be preserved.

**Fig. 3:**
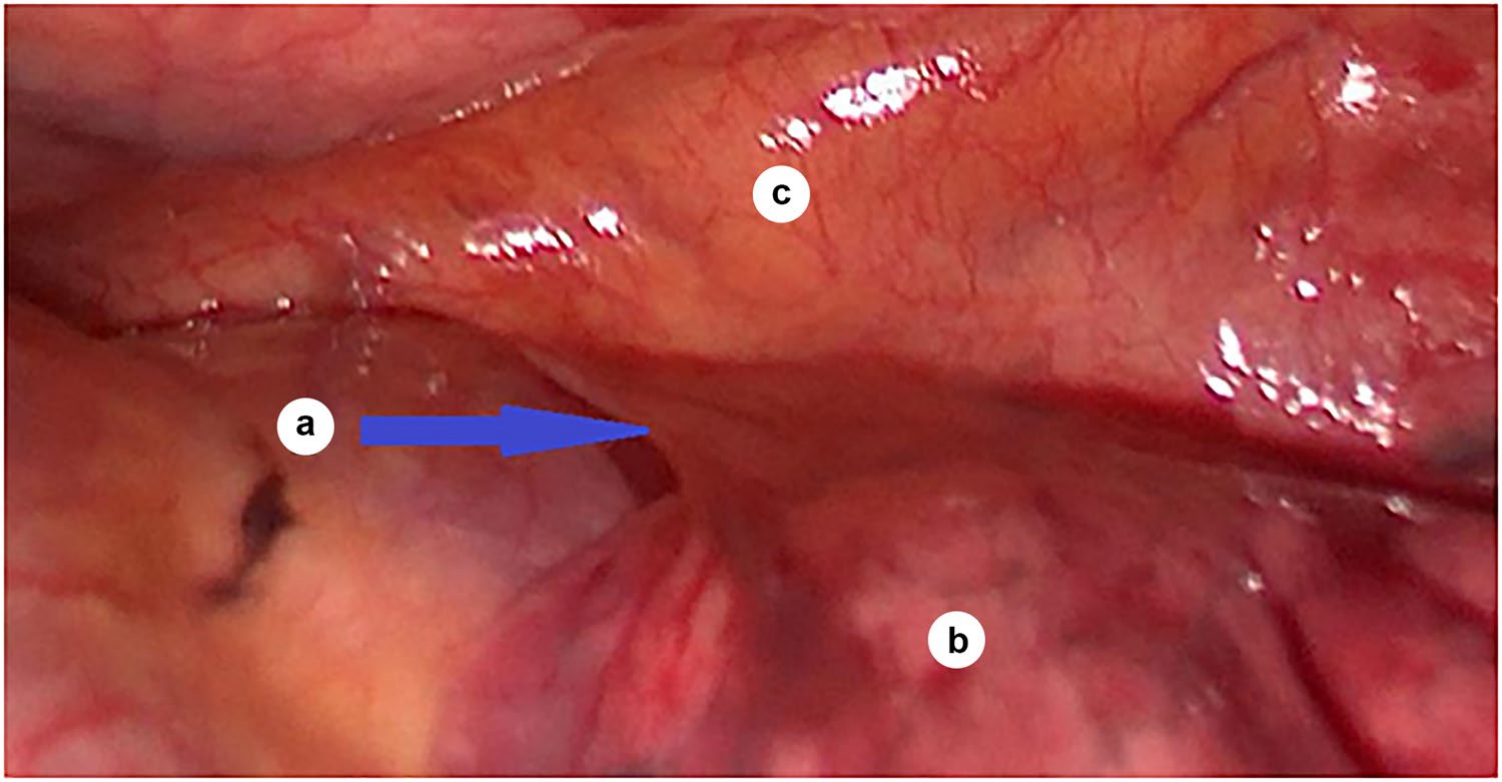
Esophagus in the area of the pulmonary ligament; *a*—pulmonary ligament; *b*—the lower lobe of the left lung; *c*—esophagus (under the mediastinal pleura)

Mobilization of the esophagus was performed with two incisions parallel to the azygos vein under the level of its arch, making the azygos vein the lateral border and the pulmonary ligament the medial border of the transection of the mediastinal pleura (Fig. 3).

Thus, the esophagus's mobilization toward the costo-diaphragmatic recess allows dissection of the lower thoracic paraesophageal lymph nodes (group N110, according to the Japanese Society for Diseases of the Esophagus classification) [10].

**Fig. 4:**
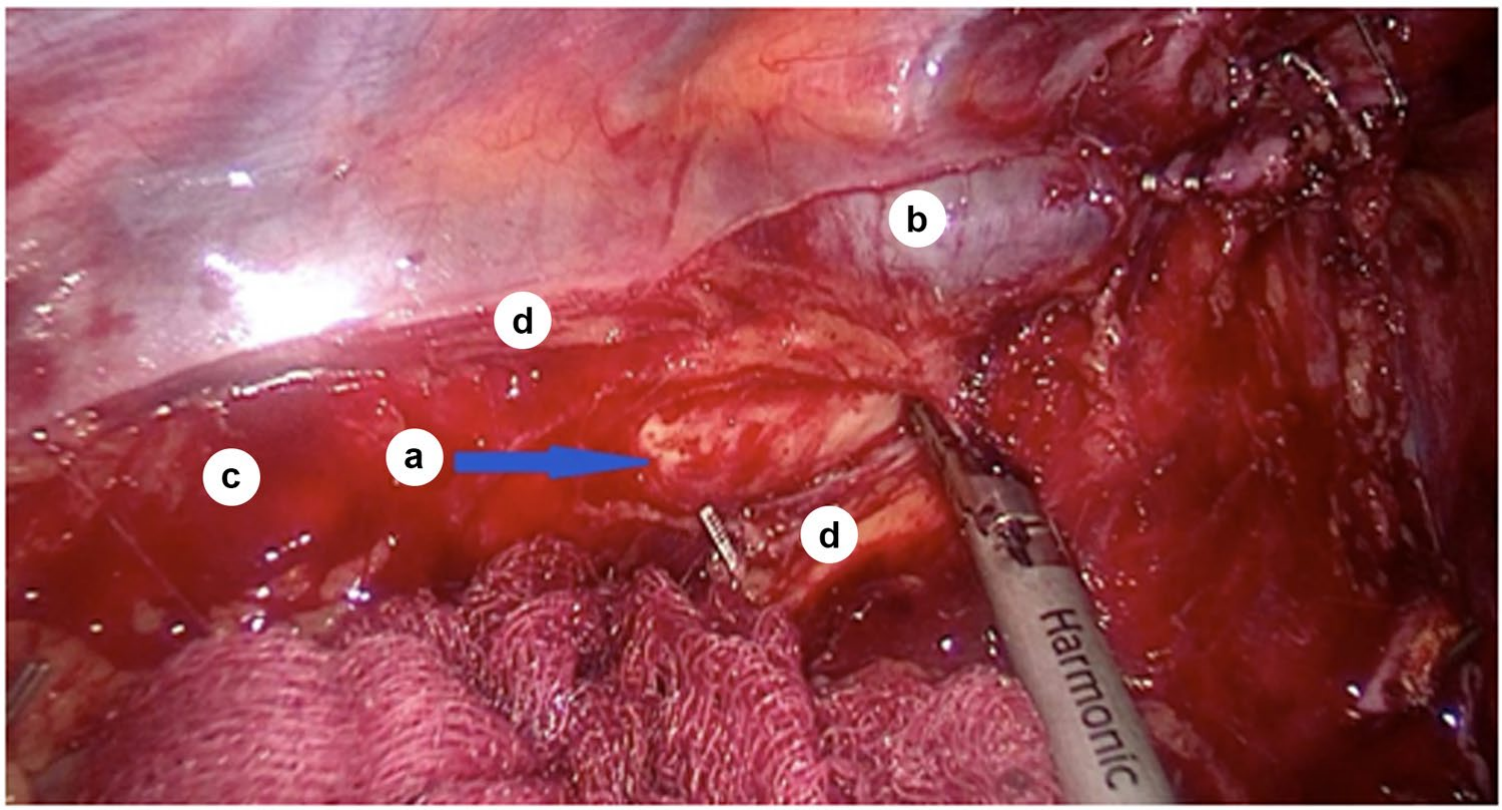
Aortic wall in sulcus azygoaortalis; *a*—aortic adventitia; *b*—azygos vein; *c*—sulcus azygoaortalis; *d*—the edge of the mediastinal pleura after dissection

Considering the lateral bending of the descending aorta and the medial bending of the esophagus, the dissection of the mediastinal pleura is performed in the groove between the azygos vein and the descending thoracic aorta. Starting at the level of the T5 vertebra, the azygos vein occludes the descending aorta and forms the azygoaortal sulcus, the area of transition of the mediastinal pleura from the azygos vein to the esophagus (Fig. 4).

**Fig. 5:**
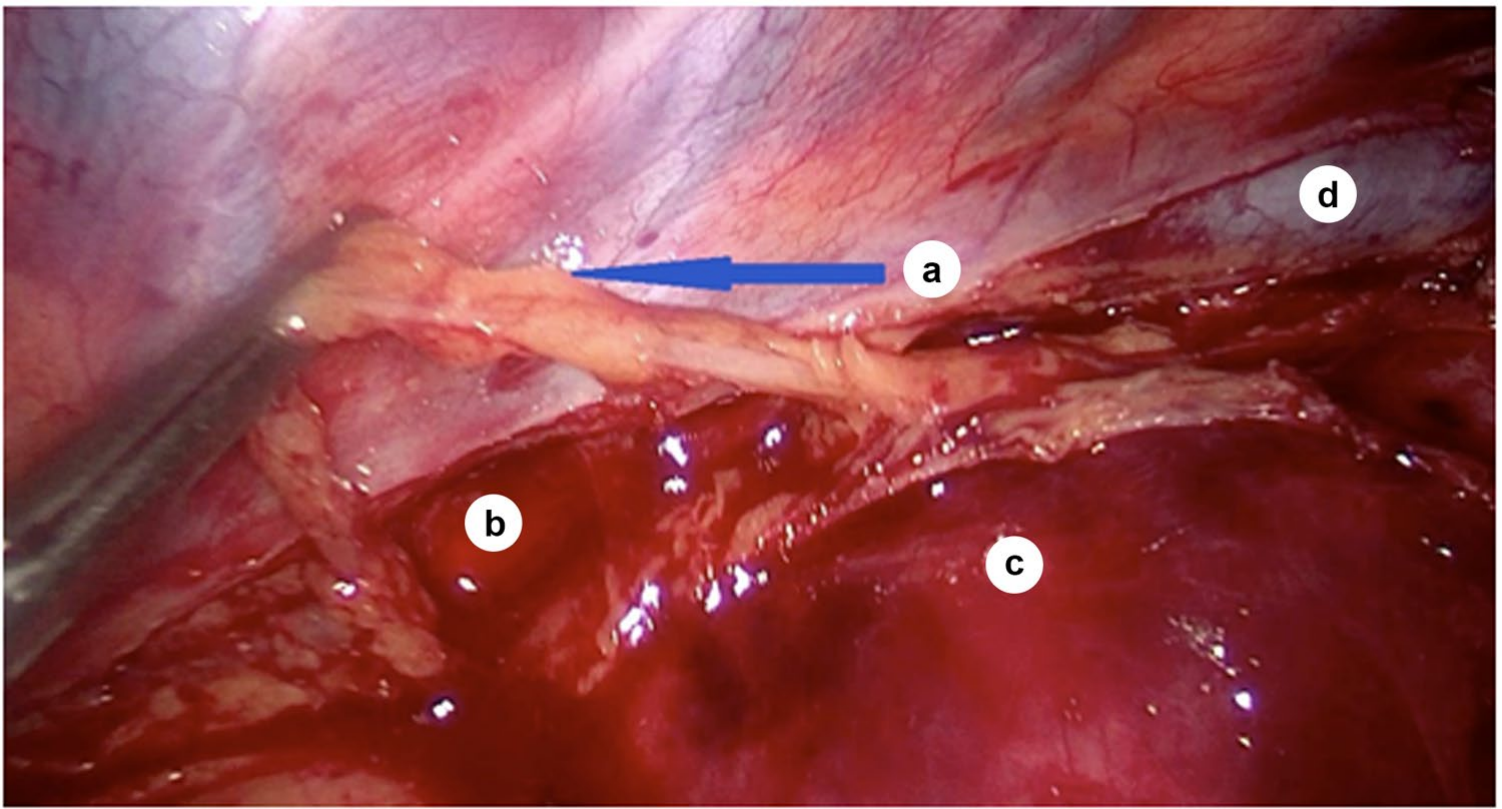
Thoracic duct (intraoperative image); *a*—thoracic duct; *b*—sulcus azygoaortalis; *c*—esophagus (under the mediastinal pleura); *d*—azygos vein

When the mediastinal pleura is opened, in the groove between the azygos vein and esophagus, the thoracic duct is entirely within the loose connective tissue, covered by the mediastinal pleura which extends from the azygos vein to the esophagus to the level approximately corresponding to the inferior pulmonary vein. Therefore, the thoracic duct was isolated and stapled as close as possible to the diaphragm, while the paraesophageal tissue was removed down to the aortic adventitia veins (Fig. 5).

The esophagus’s mobilization is continued cranially from the diaphragm, and the posterior mediastinal lymph nodes (group N 112), located within the pulmonary ligament, were moved toward the detachable specimen. The pulmonary vein, corresponding to the level of the T7 vertebra and the inferior border of the right pulmonary root, serves as the upper limit of mobilization of the pulmonary ligament. The largest of the esophageal branches of the thoracic aorta, located at the level of the T8 vertebra, is stapled when necessary. The inferior pulmonary vein can also serve as a reference point for determining the azygoaortic sulcus level, where the groove no longer passes between the azygos vein and the esophagus as from this point, it lies between the azygos vein and the aorta. After transection of the pulmonary ligament, the pericardium becomes directly visible.

The esophagus becomes mobile after transection of the ligamentum pulmonale. Two parallel incisions on the mediastinal pleura contouring the esophagus were joined at the caudal diaphragmatic segment, where the diaphragmatic lymph nodes (group N111) were located along with the surrounding areolar tissue.

At the lung's hilum, in the projection of the right and left main bronchi, subcarinal (group No. 107), and the main bronchus (group N109), lymph nodes were removed. Damage to the aorta is unlikely, because the esophagus is located on the right main bronchus, whereas the aorta is located above the esophagus.

After removing the lymph nodes, the vagus nerve becomes visible along the esophageal wall at the tracheal junction level, where it gives off branches to both lungs’ upper lobes. Preserving these branches of the vagus nerve prevents the lungs’ denervation, which could complicate the postoperative period. In addition, the vagus nerve, located caudal to the tracheal bifurcation, can be transected, because it has already delivered its bronchial branches. At the same time, its presence creates additional stiffness for mobilization of the esophagus.

In the brachiocephalic trunk region, the right laryngeal recurrent nerve branching from the vagus nerve is visible in the tracheoesophageal sulcus and must be accurately identified and spared; manipulations near this nerve should not be performed with heated instruments.

Identifying the recurrent laryngeal nerve allows identification and removal of the upper thoracic paraesophageal lymph nodes (group N105), located above this nerve, and the right recurrent laryngeal nerve lymph nodes (group 106 recR), which are located below it.

The esophagus was mobilized entirely along with the lymph nodes and surrounding adipose tissue. Thus, the inferior thyroid artery can be considered the upper limit of mobilization of the thoracic esophagus.

## Results

### Phase 1 (before opening of the mediastinal pleura)

#### Azygos vein

In all observations, during examination in the right pleural space, the first and most prominent landmark was the azygos vein, which lay along the spine and was covered by the mediastinal pleura, through which it was visible. It ran dorsal to the esophagus’s right edge and was situated on the right side of the descending thoracic aorta. At the 4th thoracic vertebra level, the azygos vein diverged sharply perpendicular to the spine, spread across the esophagus and right main bronchus, and drained into the superior vena cava, forming an arch of the azygos vein, which was one of the most stable landmarks.

The height of the arch of the azygos vein might vary in different individuals, as shown below (Table 1).

**Table 1:** Vertebral level of the arch of the azygos vein

Level	% male of cases	% of female cases
T2–T3	8.33	11.11
T3	16.67	11.11
T3–T4	25.00	16.67
T4	50.00	61.11

The most frequent overall level of the arch of the azygos vein was T4 (56.7%), followed by T3–T4 (20.0%), levels T3 (13.3%), and T2–T3 (10%) were less frequent, without significant difference between the genders.

Laterally of the esophagus, in the frontal plane, starting from the lung’s root, the pulmonary ligament was seen, which was a double layer of the mediastinal pleura and descended toward the diaphragm.

#### Vagus and recurrent laryngeal nerve

Further examination of the thoracic cavity caudal to the azygos arch revealed the vagus nerve under the mediastinal pleura adjacent to the esophagus. It ran medially to the ascending portion of the azygos vein. At the tracheal bifurcation level, the vagus nerve’s bronchial branches branched off to both lungs’ upper lobes, which were preserved to avoid denervation of the lung.

In the superior mediastinum beneath the mediastinal pleura, the right vagus nerve is visible, lying along the dorsal surface of the superior vena cava lateral to the esophagus, along the surface of the trachea, and anterior to the right subclavian artery at the site of its origin from the brachiocephalic trunk. The vagus gives off the right recurrent laryngeal nerve at this site, which passes into the tracheoesophageal sulcus.

We examined the level of the loop of the right recurrent laryngeal nerve over the subclavian artery (Table 2).

**Table 2:** Vertebral level of the loop of the right recurrent laryngeal nerve over the right subclavian artery

Level	% male of cases	% of female cases
T1	50.00	50.00
T1–T2	16.67	27.78
T2	25.00	16.67
T2–T3	8.33	5.56

As follows from the table, in our specimens, the overall most common vertebral level of the loop of the right recurrent laryngeal nerve was T1 (50.0%), followed by T1–T2 (23.3%) and T2 (20.0%), while the level of T2–T3 (6.7%) with no significant difference between genders.

In surgical procedures, the vagus nerve was distinguished from the phrenic nerve, which lay anterior and lateral to the vagus nerve adjacent to the superior vena cava's lateral surface. In contrast, the vagus nerve ran along the posterior surface of the superior vena cava. Thus, the right vagus nerve and the phrenic nerve could be identified by their different relationship to the superior vena cava.

#### Esophagus

The esophagus could be easily localized because of its relationship to the vagus nerve, running immediately along the esophageal wall. In the upper mediastinum at the level of the T4 vertebra, the esophagus was located closer to the left side of the spine. At the level of the T4–T5 vertebrae, the esophagus crossed the aortic arch and deviates to the right. At the level of the T7 vertebra, it began to deviate to the left again and crossed the anterior surface of the aorta.

In the lower thoracic region, the esophagus was seen located below the arch of the azygos vein. Starting at the tracheal bifurcation level and extending approximately to the inferior pulmonary vein level, the mediastinal pleura extended between the esophagus and the azygos vein. From the level of the T5 vertebra, the azygos vein encircled the descending aorta and formed the azygoaortic sulcus.

### Phase 2 (after opening of the mediastinal pleura)

**Fig. 6:**
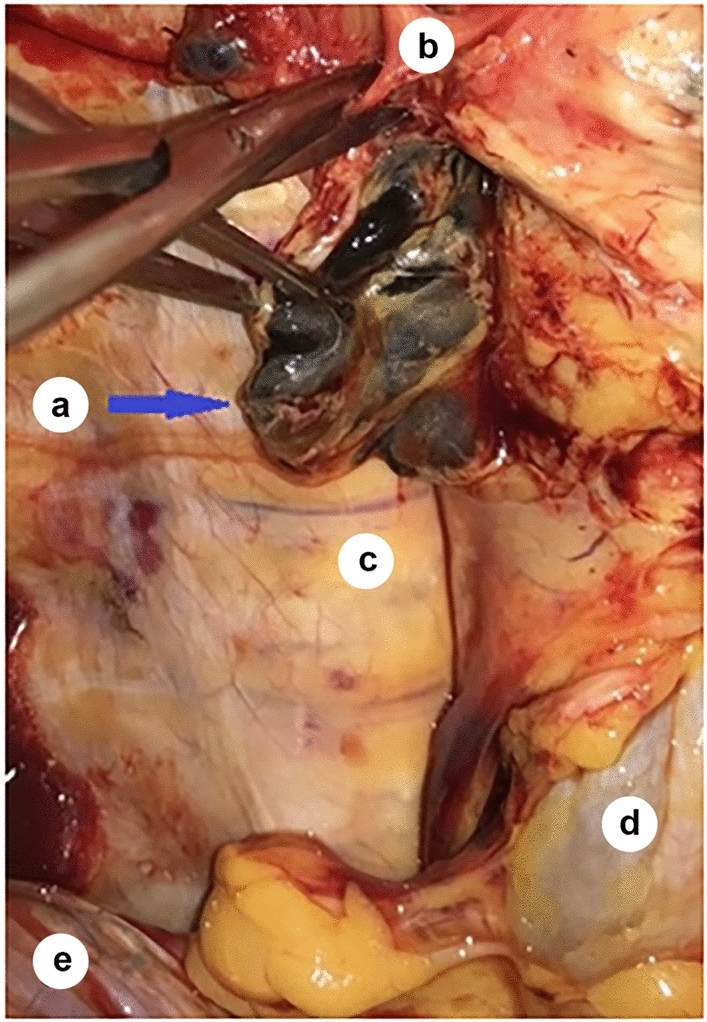
Tracheal bifurcation, front view (after the trachea has been retracted aside); *a*—subcarinal lymph nodes; *b*—branches of the vagal nerve; *c*—T7 vertebra; *d*—esophagus; *e*—the diaphragm

Transection of the azygos arch followed by sharp dissection of the mediastinal pleura and penetration into the posterior mediastinum allows optimal access to the tracheal bifurcation, the main bronchi, and thus to the right and left tracheobronchial angles, where the subcarinal lymph nodes (group N107) and the lung hilum lymph nodes (group N109) are located (Fig. 6).

The following anatomic landmarks were exposed after the mediastinal pleura was opened.

#### Bronchial artery

At the level of the tracheal bifurcation’s inferior surface, the right bronchial artery approaches the esophagus. We evaluated the number of bronchial arteries in our specimens; the results are shown below (Table 3).

**Table 3:** Number of the bronchial arteries

Number of arteries	Number of cases	%
1	1	3.33
2	7	23.33
3	13	43.33
4	8	26.67
5	1	3.33
Total	30	100

As follows from Table 3, the most common number of bronchial arteries was 3 (43.3%), followed by 4 (26.7%) and 2 (23.3%). Thus, although not entirely common (3.3%), the only bronchial artery's presence requires prudent management.

#### Pulmonary ligament and inferior pulmonary vein

In the region of the upper edge of the pulmonary ligament corresponding to the T7 vertebra, the inferior pulmonary vein was seen. In addition, at the level of the distal part of the pulmonary ligament corresponding to the T7 vertebra, the largest of the oesophageal branches of the thoracic aorta were identified.

Above the arch of the azygos vein, the right pleural sac approached the trachea's lateral wall, forming an anatomical space containing paratracheal lymph nodes (group N106). Here, the pleura was slightly separated from the trachea. In the paraesophageal adipose tissue, there were mid-thoracic paraesophageal lymph nodes (group N108). Right and left paratracheal lymph nodes were located in the pretracheal adipose tissue (groups R106 and L106, respectively). Traction of the esophagus towards linea bispinalis opened access to the aortopulmonary window and a group of its lymph nodes (left paratracheal lymph nodes, group L106) located in the area of the aortic arch, behind the esophagus.

#### Sulcus azygoaortalis

**Fig. 7:**
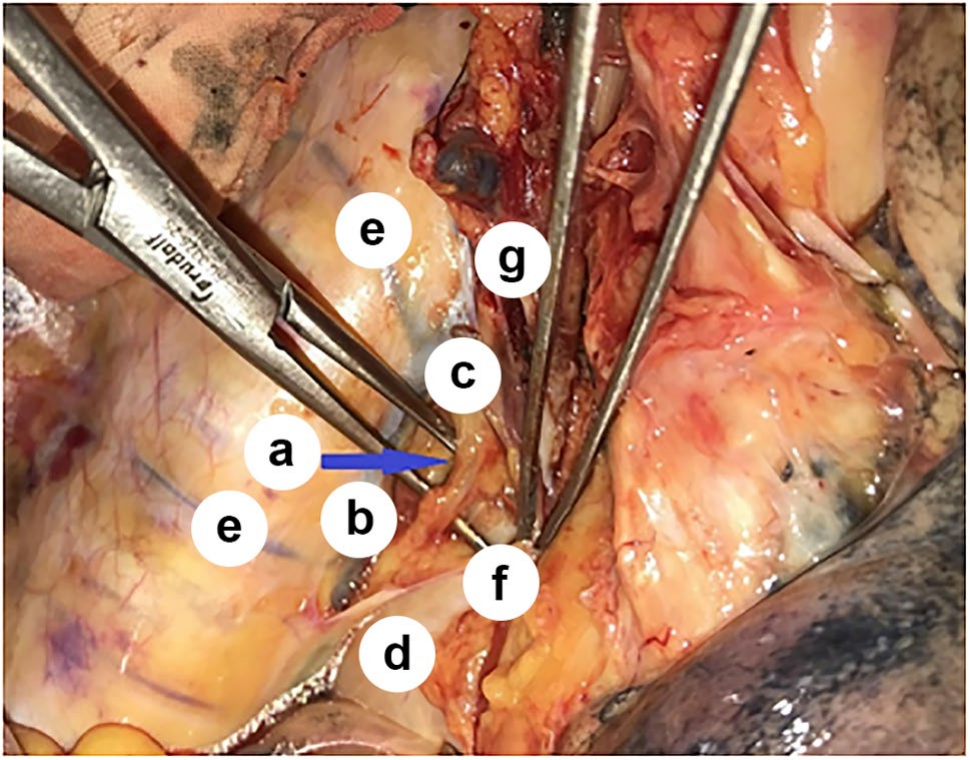
Relations between the azygos vein and the thoracic duct; *a*—thoracic duct (shown on the branches of the dissector); *b*—azygos vein; *c*—arch of the azygos vein; *d*—esophagus (under the mediastinal pleura); *e*—intercostal veins; *f*—mediastinal pleura; *g*—branches of the vagal nerve

Transection of the mediastinal pleura along the edge of the azygos vein caudal to the costodiaphragmatic recess allows access to the middle thoracic paraesophageal lymph nodes (group N108). Opening the mediastinal pleura from the level of the T4 vertebra, below the level of the arch of the azygos vein, where the mediastinal pleura reflected from the azygos vein to the esophagus and formed the azygoaortic sulcus, allowed access to the thoracic duct along its length, which was clamped intraoperatively to prevent postoperative lymphatic congestion (Fig. 7).

In the middle and lower part of the posterior mediastinum, the lower thoracic paraesophageal lymph nodes (group N110) were seen in the adipose tissue lateral to the esophagus. At the same time, medial to the esophagus–diaphragmatic and posterior mediastinal lymph nodes (groups N111 and 112, respectively) could be identified.

Transection of the pulmonary ligament toward the root of the right lung and the inferior pulmonary vein allowed visualization of a group of posterior mediastinal lymph nodes (group N112) and the pericardium, whose projection corresponded to the inferior pulmonary vein. The inferior pulmonary vein could be seen at the upper edge of the pulmonary ligament (level of T7 vertebra), which was the lowest pulmonary root component.

Transection of the pulmonary ligament toward the root of the right lung and the inferior pulmonary vein allows visualization of a group of posterior mediastinal lymph nodes (group N112) and the pericardium, whose projection corresponds to the inferior pulmonary vein. The inferior pulmonary vein can be seen at the upper edge of the pulmonary ligament (level of T7 vertebra), the lowest pulmonary root component.

Above and dorsal to the bifurcation of the trachea and right main bronchus, the esophagus was closely related to the trachea and formed the esophageal–tracheal groove in which the right recurrent nerve ascended. To mobilize the right recurrent laryngeal nerve, a sharp transection of the mediastinal pleura was performed in a cranial direction above the right vagus nerve, which lay lateral to the esophagus on the dorsal surface of the superior vena cava. The right recurrent laryngeal nerve meanders under the right subclavian artery from anterior to posterior. Therefore, visualization of the recurrent laryngeal nerve was crucial for avoiding its lesion.

Above and below the right recurrent laryngeal nerve are upper thoracic paraesophageal (group N105) and right recurrent laryngeal lymph nodes (group recR N106), respectively. At the branch of the right recurrent laryngeal nerve from the vagus nerve under the adipose tissue layer, the brachiocephalic trunk and the right subclavian artery's initial part can be found, which lies between the trachea and the superior vena cava. In addition, the brachiocephalic truncus originating from the aortic arch is visible above the upper border of the thoracic trachea in the region of the superior aperture. At this level, the pretracheal tissue contains right paratracheal lymph nodes (group R106).

The described topographic and anatomic landmarks used in sequential video-assisted navigation in the mediastinum were significant for improving the accuracy of the surgical technique and further elaboration of the technical aspects of surgical intervention for the extirpation of the esophagus designed to reduce the number of intra- and postoperative complications, especially in the prone position of the patient.

## Discussion

Surgeons increasingly prefer minimally invasive techniques in esophageal cancer’s surgical treatment, increasing the importance of surgical anatomy [3, 5]. In addition, it is vital to consider anatomical variations of the esophagus and its associated structures and visualize the supplying vessels and nerves intraoperatively, as their damage may lead to hemorrhage and impaired respiratory function in the postoperative period [17, 27]. Insufficient knowledge of mediastinal anatomical landmarks can lead to severe intraoperative complications, including death, when, for example, during the opening of the mediastinal pleura in the area of sulcus azygoaortalis, the heated branch of an electrosurgical instrument inadvertently touches the outer surface of the aorta, causing the fatal thermal injury of its lateral surface.

Our study showed that one of the most important anatomical landmarks was the azygos venous arch. At the same time, its vertebral level is subject to variation from the T2 level to the T5 level [13, 20, 24, 30]. According to our data, its most frequent location is at the T4 level (56.7%), which is consistent with the other authors' data (52.8%) [20], and slightly different from the data of the investigators, who showed that this level corresponds more frequently to the T5 level [24]. Furthermore, it has been demonstrated that the azygos vein joins the superior vena cava at the T3 level in 62.5% [13] and 53.33% [18]**,** while the arch of the azygos vein and its confluence with the superior vena cava were at the same level [20].

In thoracoscopic procedures, it is advisable to use the arc of the azygos vein alternatively to determine the level of the 4th thoracic vertebra and the provisional boundary between the superior and posterior mediastinum.

Proper intraoperative visualization of the arch of the azygos vein is of utmost importance. In addition, considering the esophagus’s curvatures in the frontal plane, the inferior pulmonary vein can serve as a reference point for determining the height of the azygoaortic sulcus, where the groove no longer runs between the azygos vein and the esophagus but between the azygos vein and the aorta.

From the findings above, we recommend the following anatomical landmarks for thoracoscopic extirpation of the esophagus in the prone position, which are suitable for direct visualization without opening the mediastinal pleura:

- The arch of the azygos vein can be used as a landmark for the upper and posterior mediastinum provisional subdivision.
- The arch of the azygos vein can also be used as a reference point for determining the height of the T4 vertebra and serves as an alternative to the external bone structures traditionally used in open surgery.
- The vagus nerve can be used as a reference point for finding the esophagus caudal to the azygos venous arch and finding the superior vena cava, trachea, and esophagus cranial to the azygos venous arch.
- The right phrenic nerve and superior vena cava can serve as additional landmarks for finding the upper mediastinum’s vagus nerve.
- The ligamentum pulmonale is a reference point for finding the inferior vena cava and thus the elements of the right pulmonary root, as well as the pericardium and posterior mediastinal lymph nodes (group N112).
- The azygos vein is a guideline for the identification of the azygoaortic sulcus.

Prior to the thoracic esophagus’s mobilization, a transection of the mediastinal pleura should be performed closer to the superior or inferior aperture, depending on the surgeon’s individual preference.

For thoracoscopic extirpation of the esophagus in the prone position, we recommend the following anatomical landmarks, which are available for direct visualization after opening the mediastinal pleura:

- the point of reference for determining the location of the thoracic duct is azygos vein and azygoaortic sulcus;
- a landmark for finding the bifurcation of the trachea, the main bronchi, the subcarinal (group No. 107) and the middle thoracic paraesophageal (group N108) lymph nodes, the esophagus, the right bronchial artery, and the aortic arch is the v. azygos arch;
- landmarks for the detection of the esophagus, subcarpal lymph nodes (group N107), middle thoracic paraesophageal lymph nodes (group N108), lung gate lymph nodes (group N109 in the area of the right main bronchus and the vena azygos) are the tracheal bifurcation and the main bronchi;
- the esophageal tracheal groove can serve as a guide for finding the right recurrent laryngeal nerve in the upper mediastinum;
- the esophagus serves as a reference point for finding the lower thoracic paraesophageal (group N110), supradiaphragmatic (group N111), and posterior mediastinal (group N112) lymph nodes which are commonly removed in patients with oesophageal cancer;
- the trachea is a reference point for finding the middle thoracic paraesophageal (group N108), right paratracheal (group R106), and left paratracheal (group L106) lymph nodes;
- the right recurrent laryngeal nerve is a reference point for finding the upper thoracic paraesophageal (group N105) and right recurrent laryngeal (group R106) lymph nodes;
- the right recurrent laryngeal nerve is a reference point for finding the brachiocephalic trunk and the right subclavian artery’s initial part.

At the level of the inferior surface of the tracheal bifurcation, the right bronchial artery approaches the esophagus; therefore, its ligation before lymphadenectomy in this area would eliminate the need to stop barely controllable arterial bleeding in a narrow subcarinal space, reducing the risk of thermal lesion of the main bronchial base.

Ligation of the bronchial artery branch exiting under the carina does not affect the bronchial blood supply. Therefore, it does not usually worsen the clinical situation during topographic and anatomic navigation. However, because the only bronchial artery that plays an essential role in blood supply to the lower part of the trachea, its bifurcation is not rare and varies from 2.5 [1] to 3.3% of the observations in the present study, bronchial arteries should be visualized intraoperatively and preserved if possible. Therefore, we support the opinion that awareness of the bronchial arterial system variations is crucial in thoracic surgery [7].

Preservation of the bronchial rami of the vagal nerves that control the cough reflex, regulate bronchial diameter, and bronchial secretion production is critical, because it reduces the likelihood of postoperative respiratory complications. Pulmonary vagotomy during esophageal extirpation may be a critical factor in developing postoperative pulmonary complications, as postoperative pneumonia is less frequent in the early postoperative period with thoracoscopic access [8, 21]. It should be considered that intraoperative injury of the vagus's bronchial branches above the tracheal junction level results in denervation of the lung. In contrast, below this level, the vagus nerve can be transected, as it has already delivered the bronchial branches, and its presence creates additional rigidity during mobilization of the esophagus.

Intraoperative removal of the paratracheal and upper thoracic paraesophageal lymph node groups in the upper mediastinum may be complicated by damaging the recurrent laryngeal nerves. Our data regarding the vertebral level of the loop of the right recurrent laryngeal nerve across the subclavian artery are consistent with other observations [2] that it is most common at the T1 vertebral level; other investigators found it more common at the T1–T2 (43%) or T2 (43%) level [15]. As our anatomic and clinical observations show, the upper mediastinum's recurrent laryngeal nerve generally has a fairly typical course. Nevertheless, we share other investigators' opinion that the anatomic variants of the recurrent laryngeal nerve cannot be predicted with absolute accuracy in advance. Therefore, an injury may occur due to incorrect visual intraoperative identification [16, 25].

Anatomic variants of the thoracic duct have been observed in 40–60% of patients [4, 11], further perplexing complex interventional procedures predisposing to iatrogenic complications. In approximately half of our observations, we also noted a relatively wide range of positional variations of its upper and lower thoracic regions relative to the left halves of the thoracic vertebrae.

## Conclusion

The use of topographic and anatomic navigation allows navigation in the posterior mediastinum’s anatomic space. Thus, it contributes to safe thoracoscopic extirpation of the esophagus with the patient in the prone position.

## Data Availability

Not applicable.
